# The relationships among MAOA, COMT Val158Met, and 5‐HTTLPR polymorphisms, newborn stress reactivity, and infant temperament

**DOI:** 10.1002/brb3.1511

**Published:** 2019-12-29

**Authors:** Zdenka Bajgarova, Adam Bajgar

**Affiliations:** ^1^ Department of Pedagogy and Psychology Faculty of Education University of South Bohemia Ceske Budejovice Czech Republic; ^2^ Department of Molecular Biology and Genetics Faculty of Science University of South Bohemia Ceske Budejovice Czech Republic

**Keywords:** 5‐HTTLPR, COMT, gene polymorphisms, HPA axis reactivity, infant temperament, MAOA

## Abstract

**Introduction:**

Variance in hypothalamic–pituitary–adrenal (HPA) axis reactivity is considered to be one of the sources of differences in infant temperament. The cortisol enters into interactions with dopamine and serotonin, so it is expected that polymorphisms in genes coding monoamine metabolism influence both HPA axis reactivity and temperament.

**Methods:**

We therefore explore the relationship among 5‐HTTLPR S/L, MAOA H/L, and COMT Val158Met polymorphisms, the stress reaction of newborn infants after a heel stick blood draw (measured by determining salivary cortisol at three time points), and temperament assessed at the age of 3 months using Rothbart's Infant Behavior Questionnaire—Revised (IBQ‐R) with a sample of 84 infants.

**Results:**

The decrease in the salivary cortisol correlated with nine primary scales and all three secondary scales of IBQ‐R. Children with a greater cortisol decrease were assessed as less susceptible to negative emotions, more extraverted, and more regulated. The polymorphisms that were observed were related both to the course of the stress reaction and to temperament. The 5‐HTTLPR S allele was connected to higher scores for Negative Emotionality and lower scores for Orienting/Regulatory Capacity. The presence of the MAOA L allele predisposed its carriers to higher scores for Negative Emotionality, lower scores for Orienting/Regulatory Capacity, and a lower decrease in cortisol. The Met allele of COMT Val158Met polymorphism was connected to a higher Positive Affectivity/Surgency and Orienting/Regulatory Capacity and a greater cortisol decrease.

**Conclusions:**

Contrary to previous studies referring mainly basal cortisol and its increase, the results of our study emphasize the importance of cortisol elimination in infant temperament. Another interesting finding was a higher cortisol increase, higher Distress to Limitations, Negative Emotionality, and Approach in MAOA LL homozygotes which are traditionally understood as more vulnerable toward early stress in developing later externalizing behavior.

## Graphical abstract

The rapidity of cortisol elimination after the stress event (a heel stick blood draw) was important in determining infant early temperament. The 5‐HTTLPR short allele and the MAOA low expressing allele predisposed their carriers to negative emotions proneness and lower regulatory capacity, the Met allele of COMT predisposed their carriers to more extraverted and regulated behavior.

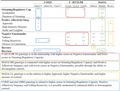

## 1 INTRODUCTION

Temperament is a highly inherited, biologically given, and relatively stable part of personality, and it manifests itself early in life (Goldsmith et al., 1987). Theoretists differ in what they include into the concept. While Thomas and Chess limit it to traits that determine formal characteristics of behavior, Goldsmith and Campos understand temperament as individual differences in the probability of experiencing, expressing and regulating the primary emotions (Goldsmith et al., 1987). Rothbart provides perhaps the most comprehensive and influential theory: It comprises of both the formal characteristics of behavior and predispositions not only to primary emotions but also to other particular responses (orienting and motor reactions). Apart from differences in behavior, she also includes differences in phenomenological experience and psychophysiological functioning into her definition. In Rothbart's words, temperament is defined as “relatively stable, primarily biologically based individual differences in reactivity and self‐regulation” (Rothbart & Derryberry, 1981, p. 40). Rothbart's first instrument for measuring temperament (Infant Behavior Questionnaire) was originally derived from nine temperamental traits resulting from the New York Longitudinal Study, to whom she added unipolar scales for fear, frustration, and positive affect. Subsequent factor analyses yielded three secondary factors of surgency, negative affect, and self‐regulation.

From a Rothbart's broad point of view, variations in threshold, latency, intensity, rise time, and the recovery of physiological responses to stress are part of the temperamental reactivity which is further modulated by self‐regulatory processes such as attention, approach, avoidance, and inhibition (Rothbart & Derryberry, 1981). While the sympathetic–adrenomedullary (SAM) system is responsible for the quick reaction to environmental challenges (the fight or flight response), HPA axis is generally considered to be crucial to the later phases of coping with stress. Cortisol, the HPA’s product, functions to increase the blood glucose level needed to supply the organism's heightened requirements while at the same time dampens the energetically demanding immune response. Cortisol is also a gene transcription factor, so it changes the expression of numerous genes that are essential to neural plasticity and therefore is considered as significant for the role of key player in the early development (Gunnar, Doom, & Esposito, 2015). Through its effects on the nervous system, cortisol changes the way the organism responds to similar stressors in the next encounter (Sapolsky, Romero, & Munck, 2000). Extreme forms of environmental challenges (loss of the parent, maltreatment) leads to a dysregulation of HPA axis function (in both direction of hypo and hyper functioning; Morris, Compas, & Garber, 2012; Tyrka et al., 2008).

The differences in both basal and reactive cortisol levels are regarded as one of the sources of temperamental variability. With regard to temperament, three higher order factors are supposed to be differently related with cortisol reactivity. In preschoolers, higher cortisol (both basal and reactive) is connected to a higher propensity to negative emotions, in particular fear (Kagan, Reznick, & Snidman, 1987; Smider et al., 2002; Talge, Donzella, & Gunnar, 2008). Counterintuitively, surgent or exuberant children have been found at times to have elevated cortisol levels (Davis, Donzella, Krueger, & Gunnar, 1999; Gunnar, Tout, de Haan, Pierce, & Stansbury, 1997). However, Gunnar (1994) hypothesized that elevated cortisol levels occur because surgent, exuberant children are much more likely to enter stressful encounters with peers and adults. Regarding effortful control in relation to basal or reactive cortisol levels, negative associations were generally found (Turner‐Cobb, Rixon, & Jessop, 2008). Even though thoroughly studied and satisfactorily explained in the preschool period, there is more ambiguity in these results of studies regarding the relationship between cortisol stress response and early temperament below three years of age. It is not clear whether a more intense cortisol reactivity is related to higher (Blair et al., 2008; Van Bakel & Riksen‐Walraven, 2004) or lower (Beijers, Riksen‐Walraven, & Weerth, 2012; Gunnar, Porter, Wolf, Rigatuso, & Larson, 1995) propensity for negative emotions or whether these variables are unrelated to each other (White, Gunnar, Larson, Donzella, & Barr, 2000).

On one hand, HPA axis reactivity is a genetically determined part of temperament, and on the other hand, HPA axis itself is largely shaped by early experiences with stress and concurrent social support (Gunnar et al., 2015), resulting in the relatively stable patterns of coping. Because of its initial setup through environmental influences, it is considered in a role of mediator between genetic predispositions, early experiences, and developmental outcomes (Gottlieb & Halpern, 2002; Pariante & Lightman, 2008). For example, in an extensive review (Bornstein, Schuppenies, Wong, & Licinio, 2006), HPA axis reactivity was placed into the role of a mediator between genetic and environmental influences and the consequent development of depression and obesity.

Besides HPA axis variability, differences in the metabolism of dopamine and serotonin are other important factors in the biological underpinnings of temperament. Nevertheless, dopamine and serotonin enter in bidirectional relations with the physiology of the HPA axis as well, so we may expect polymorphisms in genes coding monoamine metabolism to influence both temperament and HPA axis.

5‐HTTLPR insertion/deletion polymorphism in a regulatory sequence of the serotonin transporter gene gives a short and long version of the gene. The short version of the gene is connected with a lowered expression of the serotonin transporter and a lowered reuptake of serotonin into the presynaptic neuron (Greenberg et al., 1999) presumably resulting in heightened HPA‐axis reactivity (Miller, Wankerl, Stalder, Kirschbaum, & Alexander, 2013), anxiety (Sen, Burmeister, & Ghosh, 2004), and depression (Caspi et al., 2003) in S carriers. In infants, there has been only one study of genetic underpinning of the HPA stress reaction; it indicates that newborns with the 5‐HTTLPR SS genotype showed a significantly higher endocrine response after a heel prick when compared with newborns with the LL or SL genotype (Mueller, Brocke, Fries, Lesch, & Kirschbaum, 2010). With regard to the relationship between 5‐HTTLPR polymorphism and infant temperament, S allele has been given into connection to negative emotions proneness (Auerbach et al., 1999), sensitivity to maternal anxiety during pregnancy (Pluess et al. (2011), and lower positive affect (Grossmann et al., 2011).

The main role of monoamine oxidase A (MAO‐A) is thought to be in degrading serotonin following its re‐uptake by the serotonin transporter from the synaptic cleft, although it is also capable of degrading both norepinephrine and dopamine. It is encoded by the MAOA gene, and the common VNTR polymorphism in a promoter region results in higher or lower enzyme activity (Sabol, Hu, & Hamer, 1998). A more active form of the MAOA gene has been given into connection to depression (e.g., Younger et al., 2005), and the low‐activity allele is considered in relation to a higher level of aggression (McDermott, Tingley, Cowden, Frazzetto, & Johnson, 2009) and antisocial behavior in men with a history of early maltreatment (Caspi et al., 2002). In infants, a less active form of the gene in the interaction with aversive events during prenatal development predicted higher levels of negative emotionality (Hill et al., 2013), and a more active genotype was connected to higher self‐regulation in girls (Zhang et al., 2011).

Catechol‐O‐methyltransferase (COMT) is one of several enzymes that degrade catecholamines (such as dopamine, epinephrine, and norepinephrine). A common functional polymorphism in the COMT gene which is the result of G to A mutation that translates into valine (val) to methionine (met) substitution at codon 158 has been shown to account for a fourfold decrease in enzyme activity (Lachman et al., 1996). COMT plays an important role in the degradation of dopamine in the prefrontal cortex, and thus, Val158Met polymorphism is believed to influence executive functions in favor of met carriers (Barnett, Jones, Robbins, & Müller, 2007; Bruder et al., 2005; with the possible implications for self‐regulatory capacity). Somewhat contradictory, the met allele has been given into relation to the ability to experience reward in the flow of daily life (Wichers et al., 2008) but at the same time also to a more intensive reaction to an aversive stimulus (Jensen, Lonsdorf, Schalling, Kosek, & Ingvar, 2009; Smolka et al., 2007) and to a lesser capacity to regulate this response (Bishop, Cohen, Fossella, Casey, & Farah, 2006). Data on infant populations showed a greater sensitivity to fear expression but at the same time an easier recovery from negative emotions in met carriers (Grossmann et al., 2011). Sheese, Voelker, Posner, and Rothbart (2009) found a higher level of positive emotions in 6‐month‐old val/met heterozygotes compared to both homozygote groups.

Differences in infant temperament fundamentally influence parental adjustment, parenting, and developmental outcomes (Kiff, Lengua, & Zalewski, 2011). To gain an understanding of early temperamental display, it is critical to pay attention to its biological and genetic underpinnings. The aim of our study was to explore the relationships between the genetic polymorphisms 5‐HTTLPR ins/del, MAOA H/L, and COMT Val158Met, the reaction of a newborn to a heelstick blood draw measured by the determination of salivary cortisol, and temperament at three months as rated by mothers in IBQ‐R.

The first partial aim of our study is to show whether newborn stress reactivity is related to the later temperament of an infant. A heelstick blood draw three days after delivery provides a unique chance to measure the reaction to a highly aversive stimulus very shortly after birth. Even though studies on this topic have already been carried out, they are just a few in number and their results are contradictory.

The second partial aim of our study is to explore the genetic dispositions of the reaction to stress in neonates as well as infant temperament. To our knowledge, there is only one study of the genetic underpinnings of newborn reaction to stress, and thus, our results are genuinely new in this respect. There are more studies of the genetic background of temperament, but they are certainly not abundant.

The third partial aim of the study was to show whether the genetic influence on temperament is mediated by newborn stress reactivity. To the best of our knowledge, our study is the first one to explore all three variables in an infant sample and thus enables us to test the hypotheses of mediation.

## 2 METHODS

### 2.1 Participants

One hundred physiological newborns and their mothers were recruited in the Department of Neonatology in the Ceske Budejovice Hospital. The research design was approved by the Ethical Committee of the Ceske Budejovice Hospital. The final sample consisted of 84 participants (56% male; 44% female); 16 participants were excluded from the sample because of data incompleteness. Because of the considerable homogeneity of the Czech population, all the participants were Caucasians. All the mothers signed an informed consent. The mothers’ demographic characteristics are summed up in Table 1.

**Table 1:** Demographic characteristics of mothers

Variable	Variable category	% of sample
Family status	Unmarried	29.6
	Married	70.4
Number of children	1	51.9
	2	36.4
	3	7.8
	4	3.9
The highest reached educational attainment level	Elementary school	13.6
	Junior high school	66.7
	High school	19.7
Size of place of residence	1–1,000	19.8
	1,000–5,000	28.4
	5,000–10,000	7.4
	10,000–50,000	1.2
	50,000–100,000	40.8
	100,000–1,000,000	1.2
	More than 1,000,000	1.2

### 2.2 Data collection and analysis

#### 2.2.1 Newborn stress reactivity

Saliva was collected in the third day of the life of a newborn (mean 69 hr) at three different time points; before a heelstick blood draw (basal cortisol), 20 min after (assumed peak of reaction), and 45 min after the heelstick blood draw (assumed time of recovery from the negative stimulus). The times of the second and third saliva collections (peak and recovery) reflect the assumed time needed for the manifestation of cortisol changes in saliva; 20 min was decided on (Lewis & Ramsay, 1995). The baby's mouth was swept with a Salivary Infant's Swab^®^ for 60 s. After completing the collection, the samples were transferred to the Laboratory of Molecular Integrative Physiology in Drosophila of the Department of Molecular Biology of the Faculty of Science of the University of South Bohemia in Ceske Budejovice. The samples were centrifuged in a refrigerated table centrifuge (1,500 ***g***, 10 min, 4°C) and then kept frozen at −80°C until assay.

Cortisol was analyzed by the second author of the publication. The samples were assayed for cortisol using a commercially available EIA kit (expanded range high‐sensitivity salivary cortisol EIA kit, Salimetrics LLC, PA, USA) without any modification to the manufacturer's protocol (Salimetrics, State College, PA). The test had average intra‐ and interassay coefficients with a variation of 3.5% and 4.2%, respectively.

Cortisol increase was defined as the difference between Peak and Basal (Peak minus Basal), while cortisol decrease was defined as the difference between Peak and Recovery (Peak minus Recovery).

#### 2.2.2 5‐HTTLPR, MAOA H/L, and COMTVal/Met polymorphisms

DNA was obtained through the collection of infant buccal cells after the third saliva collection. The experimenter rubbed the inside of the infants’ inner cheek and gums for at least 20 s with a Cambio Catch‐All™ Collection Swab for Remote Testing. The samples were stored frozen at −80°C until assay. Genomic DNA was extracted from the cheek swab samples by standard proteinase K digestion and chloroform extraction.

Particular variable parts were amplified from gDNA samples using specific pairs of primers (5′‐GGCGTTGCCGCTCTGAATGC‐3′ and 5′‐GAGGGACTGAGCTGGACAACCCAC‐3′ for 5‐HTTLPR genotypes; 5′‐CAGCGCCCAGGCTGCTCCAGAAAC‐3′ and 3′‐GGTTCGGGACCTGGGCAGTTGTGC‐5′ for MAOA genotypes; 5′‐CGAGGCTCATCACCATCGAGATC‐3′ and 3′‐CTGACAACGGGTCAGGAATGCA‐5′ for COMT genotypes) and polymerase chain reaction (PCR; T3000 cycler; Biometra). We used 50 ng of gDNA template, gene‐specific Fwd and Rev primer in a concentration of 100 nM, and a mastermix containing a buffer, polymerase, and nucleotides (PPP MaterMix; TopBio) placed into a PCR with a volume of 50 µl. The PCR amplification protocol included denaturation at 94°C for 3 min, followed by 35 cycles at 94°C for 20 s, 62°C for 30 s, and 72°C for 30 s, followed by elongation at 72°C for 5 min. The resulting fragment length was identified with 2.5% agarose gel electrophoresis (Serva). Ethidium bromide was used for visualization of the DNA fragments. The lengths of the fragments were derived using an appropriate marker (1 kb plus, New England Biolab). The sequential correctness of representative fragments was verified by isolating fragments from the gel and subsequent sequenation (performed by the SeqMe company).

Polymorphisms that are characteristic of variable numbers of the repeated insert (MAOA) or of the presence of sequence insertion (5‐HTTLPR) were identified directly only by gel electrophoresis. RFLP (restriction fragment length polymorphism) was used to identify alleles carrying single‐base polymorphism. A pre‐amplified DNA fragment was broken using a restriction enzyme. A to G substitution in a 5‐HTTLPR variant containing insertion was recognized by the restriction enzyme MspI (New England Biolab, according to Wendland, Martin, Kruse, Lesch, & Murphy, 2006). The Val and Met alleles of COMT polymorphism were discriminated by the restriction enzyme NlaIII (New England Biolab; Lajin & Alachkar, 2011). Restriction was followed by agarose gel electrophoresis and visualization by ethidium bromide staining. The rightness of the representative fragments was verified by sequenation (SeqMe).

MAOA and COMT genotypes were distributed according to Hardy–Weinberg equilibrium; MAOA using the most frequent 3‐repeat and 4‐repeat alleles: *χ* ^2^(1, *N* = 84) = 1.81, *p* = .238; COMT Val158Met using val and met alleles: *χ* ^2^ (1, *N* = 84) = 0.43, *p* = .512. Concerning 5‐HTTLPR polymorphism, at first a short and long versions were differentiated. The genotype frequencies for the 5‐HTTLPR polymorphism based on the biallelic model (insertion/deletion) were in Hardy–Weinberg equilibrium, *χ* ^2^(1, *N* = 84) = 1.40, *p* = .244. In a long version of a gene single nucleotide, A to G substitution was considered afterward. When the biallelic model was reclassified into a triallelic model (L_A_, L_G_, and S), the resulting genotype distribution for the 5‐HTTLPR polymorphism based on the triallelic mode was in Hardy–Weinberg equilibrium: *χ* ^2^(3, *N* = 84) = 11.58, *p* = .226. Since the G allele is characterized by a lowered transcription compared to the A allele and is phenotypically closer to a short allele of a gene (Praschak‐Rieder et al., 2007), the triallelic model (L_A_, L_G_, and S) was finally reclassified to a biallelic model with the low‐expressing S and L_G_ alleles designated S′ and the higher‐expressing L_A_ allele designated L′. The resulting genotype distribution was in Hardy–Weinberg equilibrium, *χ* ^2^(1, *N* = 84) = 0.39, *p* = .512. The procentual distribution of the allele combinations of the observed polymorphisms is shown in Table 2.

**Table 2:** Distribution of allele combinations of observed polymorphisms in percent

5‐HTTLPR S/L		5‐HTTLPR A/G		5‐HTTLPR S´/L´		MAOA H/L		COMT val/met	
SS	3.53	AA	30.59	L´L´	30.59	HH	61.18	VV	24.71
SL	41.18	GG	2.35	S´L´	45.88	HL	30.59	VM	52.94
LL	55.29	SS	3.53	S´S´	23.53	LL	8.24	MM	22.35
		AG	22.35						
		SA	23.53						
		SG	17.65						

#### 2.2.3 Infant temperament at three months

Rothbart's Infant Behaviour Questionnaire (IBQ–R; Gartstein & Rothbart, 2003) was translated into the Czech language and administered to mothers when their infants were three months old. The IBQ‐R represents a 191‐item parent‐report instrument derived from Rothbart's definition of temperament (Rothbart, 1981). It yields 14 scales which, in turn, form three over‐arching factors that were identified through factor analyses: Positive Affectivity/Surgency (Activity Level, Approach, High Intensity Pleasure, Perceptual Sensitivity, Smiling and Laughter, and Vocal Reactivity), Negative Emotionality (Distress to Limitations, Fear, Sadness, and negatively loading Falling Reactivity), and Orienting/Regulatory Capacity (Cuddliness/Affiliation, Duration of Orienting, Low Intensity Pleasure, and Soothability). The Cronbach's alpha for each of the IBQ‐R scales in the present sample ranged from 0.69 to 0.85 (missing values were replaced using the item mean substitution method; see Huisman, 2000) and was generally similar to the alpha values reported by Gartstein and Rothbart (2003). Table 3 presents the descriptive statistics of the IBQ‐R scales and salivary cortisol.

**Table 3:** Descriptive statistics of IBQ‐R scales and salivary cortisol values

Scale	Mean	*SD*	Min	Max
Activity level	3.73	0.83	2.08	5.07
Distress to limitations	3.34	0.88	1.93	5.5
Fear	2.3	0.88	1.25	4
Duration of orienting	3.94	1.13	2.4	6.2
Smile and laughter	4.13	1.01	2.5	5.56
High intensity pleasure	4.98	1.12	2.5	7
Low intensity pleasure	4.83	0.93	2.86	7
Soothability	5.02	0.63	3.28	6.33
Falling reactivity	5.05	0.84	2.92	6.23
Cuddliness/Affiliation	5.89	0.58	4.5	6.75
Perceptual sensitivity	3.2	1.33	1.25	5.6
Sadness	3.57	0.94	1.92	5.18
Approach	3.8	1.2	1.88	6
Vocal reactivity	3.17	0.98	1.5	5.29
Positive affectivity/Surgency	3.83	0.7	2.58	5.25
Negative emotionality	3.1	0.85	1.99	4.62
Orienting/Regulatory capacity	4.95	0.54	3.93	5.78
Basal (µg/dl)	0.82	0.38	0.24	1.69
Peak (µg/dl)	1.41	0.42	0.45	2.58
Recovery (µg/dl)	1.12	0.44	0.16	2.07
Increase (µg/dl)	0.59	0.38	0.03	2.12
Decrease (µg/dl)	0.29	0.34	−0.21	1.61

#### 2.2.4 Missing values

All the children whose mothers did not fill in IBQ‐R (16 children) were excluded from further analysis. Neither those children nor their mothers differed from the rest of the group in their demographic characteristics. The final sample consisted of 84 newborns (56% boys, 44% girls). In 15 of these 84 children, cortisol data are missing because the amount of saliva needed for salivary cortisol analysis that was collected was insufficient.

#### 2.2.5 Statistical analyses

Due the non‐normality of some IBQ‐R scores and cortisol values and small N, nonparametric statistical methods (Spearmen's correlations, Kruskal–Wallis test) were used when possible. The post hoc analysis of Kruskal–Wallis test was carried out through the use of Mann–Whitney tests with Bonferroni correction. We consider results with p smaller than 0.05 statistically significant.

### 2.3 Controlling for a variable

#### 2.3.1 Cortisol values

**Figure 1:**
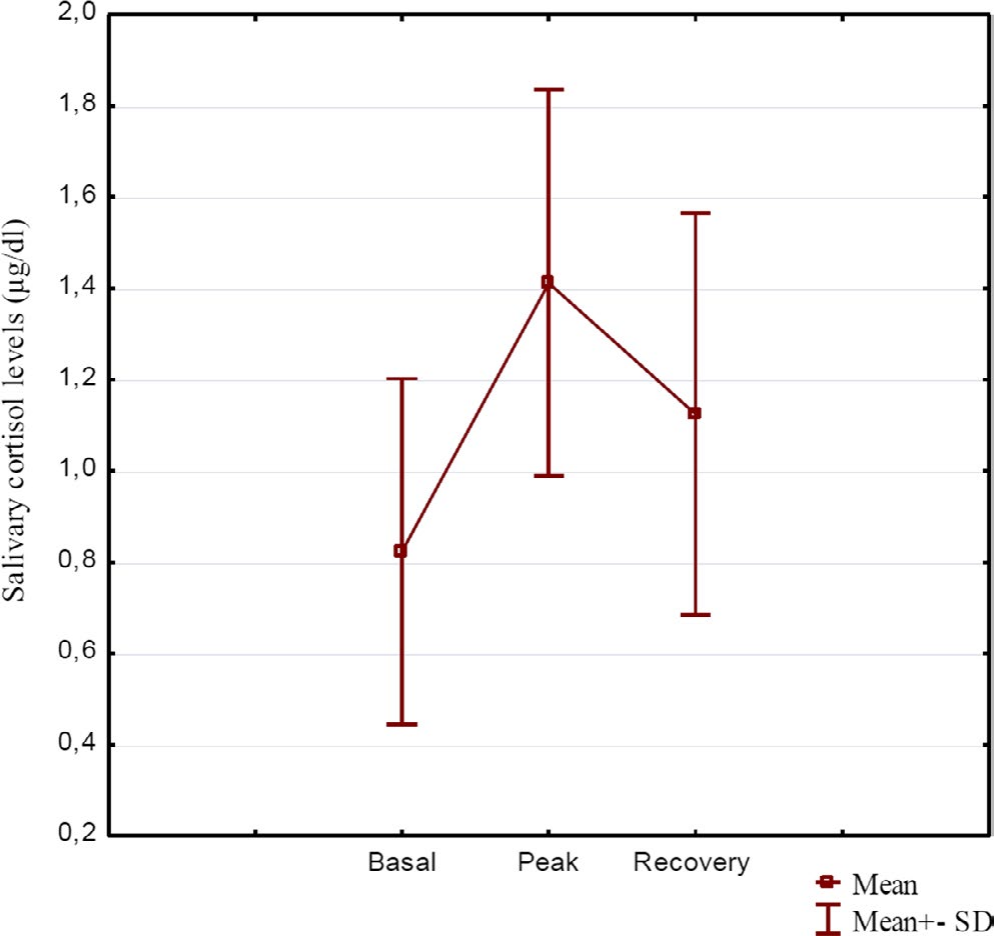
The course of HPA axis reaction; this figure illustrates means and standard deviations of salivary cortisol levels before (Basal), 20 min after (Peak), and 45 min after (Recovery) heelstick blood draw

Saliva was collected an average of 69 hr after delivery, and the age of the infants did not correlate with any of the cortisol measures (all *p* > .228). The procedure was performed in the morning hours, and since cortisol diurnal rhythm is not established at this early age (Santiago, Jorge, & Moreira, 1996), the time of the procedure was not considered as a variable. Before the first saliva collection, 72.6% of the children were sleeping and 27.4% were awake. These two groups did not differ statistically in any of the cortisol values (all *p* > .122). 11 infants (13%) were born by Caesarean section. They did not differ significantly from vaginally delivered babies in any of the cortisol measures (all *p* > .370). The gender of the child was not related to the course of stress reaction (all *p* > .133). Figure 1 shows means and standard deviations of salivary cortisol before, 20 min after, and 45 min after the heelstick blood draw.

#### 2.3.2 IBQ‐R scales

None of the IBQ‐R scales was related to a child's gender (all *p* > .087) and the mother's level of education (all *p* > .069). Size of place of residence did not correlate with the IBQ‐R scales (all *p* > .139). Single mothers rated their children as being more vocally reactive (*N* = 84; *Z* = −2.27, *p* = .024); none of the other IBQ‐R scales was related to a mother's marital status. The mothers’ age correlated with Smiling and Laughter (ρ = 0.22, *p* = .05); older mothers rated their children as showing more of these positive displays. The number of children was related to Vocal Reactivity (H [3, *N* = 76] = 10.42, *p* = .015); first‐time mothers rated their children as being higher in Vocal Reactivity (*p* after Bonferroni correction *p* = .021). One possible explanation of this trend is that a mother pays more attention to her first child and thus is more likely to register her infant's vocalization or induce it in mutual interaction.

## 3 RESULTS AND DISCUSSION

### 3.1 The relationship among newborn stress reactivity and temperament at three months

**Figure 2:**
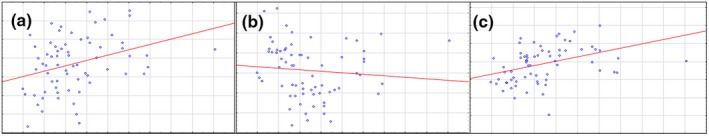
Scatter plots of Spearmen's correlations between cortisol decrease and (a) Positive Affectivity/Surgency (*r* ^2^ = .11; *p* < .01), (b) Negative Emotionality (*r* ^2^ = .07; *p* < .05), (c) Orienting/Regulatory Capacity (*r* ^2^ = .23; *p* < .001). Cortisol decrease is on x‐axis, temperament variables on *y*‐axis

Table 4 presents the Spearman correlations (ρ) between salivary cortisol values and IBQ‐R scores together with the statistical significance of a relationship. Basal cortisol correlated negatively with Activity Level; the lower the basal cortisol after delivery, the higher the Activity Level as seen by mothers. Increase and Peak did not correlate with any of the IBQ‐R scales, Recovery correlated negatively with High Intensity Pleasure and Falling Reactivity and positively with Negative Emotionality and Distress to Limitations. The decrease in salivary cortisol after the heelstick blood draw correlated with nine primary scales and all three secondary scales. Children with a greater cortisol decrease were assessed as less susceptible to negative emotions, more extraverted, and regulated. Figure 2 offers simplified scatter plots of the relationships between cortisol decrease and Positive Affectivity/Surgency, Negative Emotionality, and Orienting/Regulatory Capacity.

**Table 4:** Spearman's correlations between IBQ‐R scales and cortisol values

IBQ‐R scale	Basal	Peak	Recovery	Increase	Decrease
Orienting/Regulatory capacity	0.07	0.08	–0.21	0.07	0.48***
Soothability	–0.15	–0.06	–0.18	0.08	0.31**
Duration of orienting	0.09	0.07	–0.17	–0.03	0.31*
Low intensity pleasure	0.08	0.12	0.00	0.09	0.27*
Cuddliness	0.03	–0.12	–0.08	–0.12	0.05
Positive affectivity/Surgency	–0.12	–0.00	–0.20	0.11	0.33**
Perceptual sensitivity	–0.06	0.06	–0.01	0.11	0.07
Approach	–0.14	–0.05	–0.18	0.01	0.29*
Vocal reactivity	0.04	0.13	–0.08	0.15	0.38**
High intensity pleasure	–0.03	–0.01	–0.29*	0.07	0.39**
Smile and laughter	0.06	0.01	–0.09	–0.02	0.30*
Activity level	–0.32**	–0.15	–0.20	0.11	0.01
Negative emotionality	–0.06	0.19	0.24*	0.19	–0.26*
Sadness	–0.08	0.08	0.18	0.16	–0.29*
Falling reactivity	–0.08	–0.14	–0.29*	–0.09	0.37**
Distress to limitations	–0.01	0.19	0.24*	0.17	–0.22
Fear	–0.08	0.12	0.06	0.12	0.00

* *p* < .05; **p < .01; ***p < .001.

In our opinion, the aforementioned findings show the extent to which the inborn characteristics of the course of the HPA axis reaction (mainly the rapidity of cortisol elimination and the effectivity of the negative feedback loop) contribute to early behavioral differences in infants. We propose that the quick decrease of cortisol after its culmination after an aversive stimulus is the part of biological predisposition to extraversion and regulation, and conversely, the slower decrease of cortisol undergrids the propensity to negative emotions.

Our assumption is rather in agreement with studies of preschool and school children in which the higher cortisol (both basal and reactive) is considered to be a symptom of HPA axis dysregulation and a source of negative emotionality and less effective self‐regulation (Dettling, Gunnar, & Donzella, 1999; Gunnar et al., 1997; Kagan et al., 1987; Pérez‐Edgar, Schmidt, Henderson, Schulkin, & Fox, 2008; Smider et al., 2002; Talge et al., 2008). In infant studies actually, the results of concurrent studies are far less clear‐cut and sometimes in contradiction with the interpretation of our results. In the only study of the relation between the reaction of a newborn infant to the stress caused by a heelstick blood draw and temperament measured by IBQ‐R (Gunnar et al., 1995), the peak correlated negatively with Distress to Limitations at six months. The authors deduced that the ability to react intensively to an aversive stimulus reflects better neurobehavioral organization. In a similar way, the results of study of Beijers et al. (2012) showed that a lower score for Negative Emotionality measured by IBQ‐R at three and six months predicted a higher cortisol reactivity at one year of age. In agreement with our assumption, we can name the results of two studies carried out on toddlers; in Gunnar et al. study (Gunnar, Larson, Hertsgaard, Harris, & Brodersen, 1992), Distress to Limitations and the negative affect in nine‐month‐old infants observed in the laboratory correlated positively with a subsequent cortisol reaction to separation from the mother, while the observed positive affect correlated negatively. Another study in which higher cortisol reactivity was related to negative emotionality was conducted on infants and toddlers in institutional care; shy children showed a higher increase in afternoon cortisol (Watamura, Donzella, Alwin, & Gunnar, 2003).

A comparison of our results is impeded by the fact that most of studies on infants, toddlers, and preschool children refer to basal cortisol or cortisol reactivity (peak and increase). As a consequence, we do not have that many results to support or contradict our hypotheses of cortisol recovery importance for temperament. Two studies referring cortisol decrease (Blair et al., 2008; Kagan et al., 1987) basically indicate that the propensity to negative emotions was related to a more pronounced HPA axis reaction (higher increase followed by higher decrease of salivary cortisol). Since we did not consider increase and decrease together in our study, it is again difficult to compare the results and interpret them in light of these two studies. Even without the relevant literature to compare, our results regarding the importance of cortisol elimination are in agreement with the assumption derived from the HPA axis physiology—a quicker elimination of cortisol leads to a quicker recovery from stress. The capacity to regulate potentially harmful prolonged cortisol activation is key for extraversion and regulation, and accordingly, the lack of this capacity undergrids propensity to negative emotionality.

### 3.2 Genetic disposition to stress reaction and temperament

**Figure 3:**
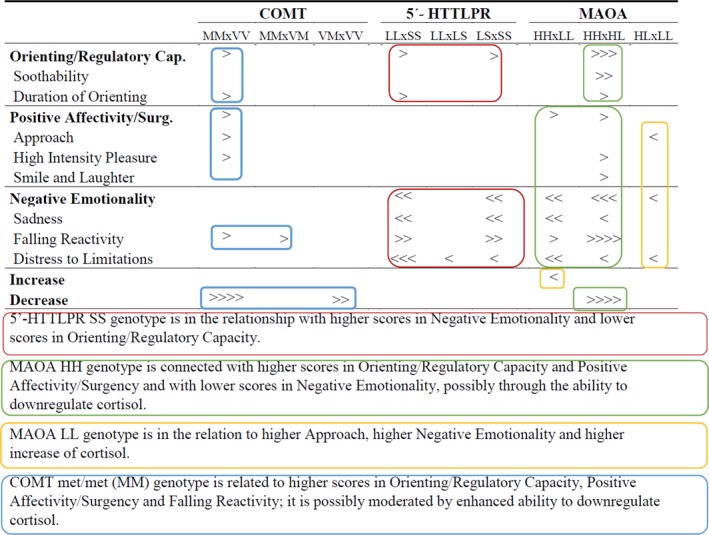
Post hoc analysis of Kruskal–Wallis test; direction of > signs denotes the direction of inequality between groups, their number denotes statistical significance of *p* after Bonferroni correction: “>”*p* < .05; “>>”*p* < .01; “>>>”*p* < .001; “>>>>”*p *< .0001. Important relationships are highlighted by colored rectangles

The Kruskal–Wallis test with two degrees of freedom was used for testing the relationships between genetic polymorphism, salivary cortisol, and the IBQ‐R scales. Tables 5, 6, and 7 summarizes test statistics (H), level of significance (p), and eta‐squared coefficients of size effects (η^2^) of statistically‐significant effects of 5‐HTTLPR, MAOA, and COMT on salivary cortisol and temperament. Since STATISTICA software does not count coefficient of size effects for nonparametric tests, Internet calculator (Effect sizes calculator for non‐parametric tests: Mann‐Whitney‐U, Wilcoxon‐W, & Kruskall‐Wallis‐H, n.d.) was used for this purpose. Figure 3 summarizes the post hoc analysis of the Kruskal–Wallis test with the use of Mann–Whitney tests with Bonferroni correction; only the differences that survived that correction are presented. The most important findings are highlighted in different colors.

**Table 5:** Effect of the 5‐HTTLPR polymorphism on temperament

	H (2, *N* = 84)	η^2^
**Orienting/Regulatory capacity**	9.10**	0.08
Duration of orienting	6.49*	0.05
**Negative emotionality**	14.22**	0.14
Sadness	12.20**	0.12
Falling reactivity	12.19***	0.18
Distress to limitations	18.04***	0.18

* *p*< .05; **p< .01; ***p< .001.

**Table 6:** Effect of the MAOA H/L polymorphism on stress reaction and temperament

	H (2, *N* = 84)	η^2^
**Orienting/Regulatory capacity**	17.10***	0.17
Soothability	10.37**	0.10
Duration of orienting	9.30	0.08
**Positive affectivity/Surgency**	8.74*	0.08
Approach	7.84*	0.07
High intensity pleasure	8.14*	0.07
Smiling and laughter	7.56*	0.06
**Negative emotionality**	23.35***	0.25
Sadness	16.08***	0.16
Falling reactivity	25.99***	0.17
Distress to limitations	16.42***	0.17

	H (2, *N* = 69)	
**Cortisol reaction**		
Increase	7.91*	0.08
Decrease	17.33***	0.22

* *p*< .05; **p< .01; ***p< .001.

**Table 7:** Effect of the COMT polymorphism on stress reaction and temperament

	H (2, *N* = 84)	
**Orienting/Regulatory capacity**	8.16*	0.07
Duration of orienting	6.66*	0.05
**Positive affectivity/Surgency**	6.67*	0.05
Approach	6.29*	0.05
High intensity pleasure	7.44*	0.06
Falling reactivity	9.36*	0.09

	H (2, *N* = 69)	
**Cortisol reaction**		
Decrease	19.32***	0.24

* *p*< .05; **p< .01; ***p< .001

5‐HTTLPR polymorphism was related significantly but with small size effects to Orienting/Regulatory Capacity and more notably to Negative Emotionality. SS homozygotes received the highest scores in Distress to Limitations, Sadness and Negative Emotionality, and the lowest score in Falling Reactivity. The connection of S allele with the propensity to negative emotionality is traditional in studies of adults (Caspi et al., 2003; Kendler, Kuhn, Vittum, Prescott, & Riley, 2005; Schinka, Busch, & Robichaux‐Keene, 2004; Sen et al., 2004), adolescents (Eley et al., 2004), children (Gibb, Uhrlass, Grassia, Benas, & McGeary, 2009), and infants (Auerbach et al., 1999; Pluess et al., 2011). Additionally, the connection of S allele with the Orienting/Regulatory scale (SS homozygotes got lower scores for Orienting/Regulatory Capacity compared to both SL and LL carriers and a lower score for Duration of Orienting compared to LL homozygotes) has been found in several infant studies. In a study of Grossmann et al. (2011), seven‐month‐old SS homozygotes had a lower score for Duration of Orienting, and in a study of Auerbach, Faroy, Ebstein, Kahana, and Levine (2001), 12‐month‐old SS homozygotes had a shorter duration of looking while playing with building blocks. Our data are also in agreement with the assumption that S carriers have got a diminished ability to shunt released serotonin from synapse; prolonged activation of neural pathways predisposes them for negative emotions propensity and lessened regulation. Surprisingly, we did not find any relation between 5‐HTTLPR polymorphism and the course of stress reaction, contrary to the conclusions of meta‐analyses of studies on adult populations (Miller et al., 2013) and the only study of newborns (Mueller et al., 2010), which both show more intensive cortisol reactivity in SS homozygotes. The missing link between 5′‐HTTLPR polymorphism and the course of stress reaction in our results also contradicts the assumption following from the role of serotonin in HPA axis activation (Cassano, & D’mello, 2001) and negative feedback control (Lowry, 2002).

MAOA polymorphism was found to exert the most robust influence on salivary cortisol and temperament in our study. It was related to all three IBQ‐R secondary scales and to both cortisol increase and decrease. HH homozygotes had the statistically lowest scores for Sadness, Distress to Limitations, and Negative Emotionality and the highest scores for Falling Reactivity compared to the HL and LL groups. At the same time, they had got higher scores for Duration of Orienting, Soothability, and Orienting/Regulatory Capacity in comparison to HL heterozygotes. HH homozygotes had got higher scores for Smiling and Laughter, High Intensity Pleasure, and Positive Affectivity/Surgency in comparison with HL heterozygotes. Lower negative emotions and higher self‐regulation in HH homozygotes that was found in our study correspond to the finding of studies at an early age (Hill et al., 2013; Zhang et al., 2011). But to the best of our knowledge, no existing study showed higher surgency in HH homozygotes seen in our study (even though the size effects were relatively smaller compared to the two other remaining secondary scales). Nonetheless, MAOA is involved in dopamine metabolism, and thus, its connection to variability in surgency may be assumed.

HH homozygotes had a statistically lower increase in cortisol than LL homozygotes and a statistically higher decrease in cortisol compared to HL carriers. Given the above‐reported role of cortisol decrease in temperament, we may assume that lower negative emotions, higher self‐regulation, and surgency in HH carriers are at least partly the result of their greater ability to downregulate cortisol after culmination of HPA stress response.

In previous research, the L allele carriers were found to be more predisposed to impulsivity and aggressiveness (Manuck, Flory, Ferrell, Mann, & Muldoon, 2000), and at the same time, more vulnerable toward aversive environmental influences in subsequent development of impulsive and aggressive behavior (Caspi et al., 2002; Eisenberger, Way, Taylor, Welch, & Lieberman, 2007; Frazzetto et al., 2007; Kim‐Cohen et al., 2006). Thus, it is of interest that in our study, LL genotype connected both higher sensitivity to aversive stimulus (higher increase of cortisol compared with HH homozygotes) and temperamental precursors of potential externalizing problems (the highest Distress to Limitations and Negative Emotionality and the higher Approach significantly compared to HL). Taken into account that very plastic HPA axis is molded by early experiences and mediates their influence on consequent development, we accordingly suggest that the HPA axis sensitivity plays an important role in the vulnerability of MAOA L carriers and that L allele predisposes them to a greater reactivity of HPA axis. L allele possession might represent the genetic background that makes HPA axis more vulnerable toward early aversive experiences. Even though the relation between the L allele and vulnerability to early adversity seems well established in the literature, the connection of L allele to HPA axis sensitivity received less scientific attention. Nonetheless, the existing research is rather in line with our results. There is no study of infant population of MAOA polymorphism's role in HPA axis. Nevertheless, the studies of children (Bouma, Riese, Doornbos, Ormel, & Oldehinkel, 2012), adult patients with chronic fatigue syndrome (Smith, White, Aslakson, Vollmer‐Conna, & Rajeevan, 2006), and male caregivers of chronically ill people (Brummett et al., 2008) showed a higher predisposition to stress dysregulation in L carriers (in agreement with our study). There is merely one study showing the opposite—Jabbi et al. (2007) found higher plasma cortisol in adult H carriers in comparison with L carriers.

COMT Val158Met was related significantly but with small size effects to Positive Affectivity/Surgency and Orienting/Regulatory Capacity and, more robustly, to cortisol decrease. Met/met carriers got higher scores for Approach, High Intensity Pleasure, and Positive Affectivity/Surgency in comparison with val/val carriers. That corresponds to a logical supposition that lower degrading of dopamine results in a higher amount of it, ultimately leading to higher extraversion. At the same time, met/met homozygotes had a higher Duration of Orienting in comparison with val/val homozygotes and higher scores for Orienting/Regulatory Capacity in comparison with val/met heterozygotes. This is in agreement with the assumption that a higher amount of prefrontal dopamine facilitates executive functions. Val/val homozygotes had the lowest cortisol decrease in comparison with the two other groups; this finding is in accordance with the role that dopamine plays in HPA‐axis negative feedback (Sullivan & Dufresne, 2006). Met/met homozygotes had statistically highest scores in Falling Reactivity. It may be logical to deduce that from all the primary scales that load to the secondary scale of Negative Emotionality COMT polymorphism was related only to Falling Reactivity, the scale that should relate the closest to cortisol elimination (prior name of a scale was Rate of Recovery from Stress, this scale had got highest correlation with cortisol decrease form Negative Emotionality scales in our data).

Besides hypothetical suppositions resulting from the role of dopamine in temperament, most of our findings summed up above concur with those of existing studies of the child population. The connection between met allele and Falling Reactivity has been found in Grossmann et al. (2011) study of seven‐month‐old infants. The relationship between COMT polymorphism and positive emotionality reported above was previously shown in a study of infants (Sheese et al., 2009). Nevertheless, in this study, six‐month‐old met/val heterozygotes had the highest positive emotionality, while in our study, met/met homozygotes had higher scores compared to val/val homozygotes. Our finding that the met allele was related to Duration of Orienting and Orienting/Regulatory Capacity in favor of met carriers is also in agreement with the existing literature. A longer attention span and lower distractibility of met carriers were shown in a study of nine‐month‐old infants (Holmboe et al., 2010). In a different study (Markant, Cicchetti, Hetzel, & Thomas, 2014), seven‐month‐old met carriers got higher scores for Duration of Orienting and higher behavioral regulation.

Our findings of a positive relationship between the met allele and Approach are in contradiction to the only study referring to significant relationships between these variables at an early age. In their already‐cited study, Markant et al. (2014) showed that seven‐month‐old val carriers reached for a new toy more quickly and had a higher Approach. In children, adolescents, and adults, results concerning this connection are mixed; in a minority of studies, the val allele indicated a predisposition to impulsivity (Eisenberg et al., 1999; Wagner et al., 2010), while in a majority, it was the met allele (Bellgrove et al., 2005; Kreek, Nielsen, Butelman, & LaForge, 2005; Paloyelis, Asherson, Mehta, Faraone, & Kuntsi, 2010). It is also possible that the ambiguity of the findings results from the intervening variable of self‐regulation. If—in compliance with our results—the met allele was a predisposition for both impulsivity and self‐regulation, the relationship between the met allele and the resulting behavior would not be direct, since the degree of control of behavior is influenced by both impulsivity and control capacity.

### 3.3 HPA axis reactivity as a mediating variable of the influence of genetic polymorphism on early temperament

In COMT and MAOA polymorphisms, all three variables (gene polymorphisms, newborn stress reactivity, and temperament at three months) were related to each other, and thus, we tested the mediating role of stress reactivity in the relationship between gene polymorphisms and temperament. We tested the assumption that the influence of COMT and MAOA polymorphism on infants’ temperament at three months is mediated by their inborn stress reactivity. We used a series of regression analysis to test this assumption according to the procedure of Baron and Kenny (1986).

The number of alleles of the polymorphisms that were examined was considered as a predictor. The decrease in cortisol was a mediator, and the IBQ‐R secondary scales were considered as a dependent variable. In MAOA H/L, the number of L alleles was not a significant predictor of a cortisol decrease (β = −0.11; *p* = .362), and thus, it was not worth continuing further with the procedure.

COMT polymorphism (the number of met alleles) had a significant influence on the cortisol decrease (β = 0.41; *p* < .0001); it has a significant influence on Positive Affectivity/Surgency (β = 0.27; *p* = .013). A cortisol decrease had a significant influence on Positive Affectivity/Surgency (β = 0.34; *p* = .004). In the simultaneous entry of both the number of met alleles and the cortisol decrease into the prediction of Positive Affectivity/Surgency, the number of met alleles became insignificant (β = 0.21; *p* = .099), while the cortisol decrease remained significant (β = 0.25; *p* = .046), and thus, Baron and Kenny's criteria were met.

We further tested the significance of indirect influence by the Sobel test (Preacher & Leonardelli, n.d.). It tests the null hypothesis that the indirect influence is not different from zero. The Sobel test revealed that the indirect effect is significantly different from zero (*Z* = 2.32; *p* = .020); the indirect influence of COMTVal158Met on Positive Affectivity/Surgency is thus significant, with a size of indirect effect c′ = 0.16.

COMT Val158Met polymorphism (the number of met alleles) had a significant influence on Orienting/regulatory capacity (β = 0.32; *p* = .003). A cortisol decrease had a significant influence on Orienting/Regulatory Capacity (β = 0.38; *p* = .001). In a simultaneous entry of both COMT Val158Met polymorphism and a cortisol decrease into regression, the cortisol decrease became insignificant (β = 0.24; *p* = .051), while the number of met alleles remained significant (β = 0.34; *p* = .006), and thus, we have to reject the mediating hypothesis.

On the basis of our finding that the size of the cortisol decrease mediated the relationship between COMT polymorphism and surgency (with a size of indirect effect c′ = 0.16), we are the first to suggest that HPA axis reactivity might mediate the influence of genetic polymorphism on infant temperament. Since the hypotheses of the mediating role of HPA axis in the relationship between genetic polymorphisms and temperament has not been tested up to now, we do not have any similar studies with which to compare. More studies focus on the mediating role of HPA axis in the relationship between early experiences with stress and support and developmental outcomes (Van den Blair et al., 2011; Bergh, Van Calster, Smits, Van Huffel, & Lagae, 2008; Yehuda et al., 2010), fewer studies consider its role in the relationship between genes and developmental outcomes (Bornstein et al., 2006; Pagliaccio et al., 2014). On the other hand, we focused on the question to what extent biological and not volitionally influenced HPA axis physiology mediates the impact of tested genes on temperamental display (only three secondary scales were tested). Even without relevant mediation studies to compare, the mediating role of a cortisol decrease is in conformity with the assumptions that arise from research studies. Met carriers have less COMT in their prefrontal cortex (Chen et al., 2004); it results in a higher release of prefrontal dopamine and its slower degrading (Yavich, Forsberg, Karayiorgou, Gogos, & Männistö, 2007), while a higher amount of prefrontal dopamine leads to the more effective termination of a stress reaction (Sullivan & Dufresne, 2006), which is a basis for extraversion (Blair, Peters, & Granger, 2004; Kontoangelos et al., 2015).

## 4 STRENGTHS AND LIMITATIONS

A strong point of our study is the fact that it was carried out at a very early infant period and thus enabled us to follow the variables that are significantly less influenced by later developed self‐regulating capacities that could complicate the relationship between genetic disposition to temperamental reactivity and its phenotypic display. Although there are studies that survey the relationship between genetic polymorphism and temperament and between salivary cortisol and temperament, there is no other study that measured all three variables at once. This allowed us to test the mediation hypotheses.

The vigor of the results presented here is limited by the self‐selection sampling strategy. Particularly in IBQ‐R data, we can expect that it could be different in those mothers who agreed to take part in the study and in those mothers who refused. However, during the statistical control of demographic variables, the relationships that were found were rather marginal.

Another limiting factor could be that the IBQ‐R questionnaire was administered at three months, when the infant's emotional displays are not fully differentiated and the lack of locomotion reduces the amount of behavior from which temperament could be inferred. For instance, we cannot yet observe fear of strangers at three months; it could have contributed to the fact that we did not find significant relationships between any variable and Fear, which is otherwise largely biologically and genetically determined, with very well observable and measurable displays.

The statistical operations with cortisol were limited because of missing data; in samples of fifteen children, we did not collect enough saliva to run the following analyses. Nevertheless, this problem is quite typical in infant salivary cortisol research (Kirschbaum & Hellhammer, 1994).

## 5 CONCLUSION AND FURTHER DIRECTIONS

In our study, we were mapping a part of the genetic and biological underpinning of early temperament. Extraversion was connected to a greater cortisol decrease and to the presence of met COMT and the high‐activity MAOA allele; negative emotionality was linked to a lower decrease in cortisol and to the occurrence of S 5‐HTTLPR and L MAOA and regulation was associated with a greater cortisol decrease and the presence of met COMT, H MAOA, and the L 5‐HTTLPR allele. We were also able to follow the genetic foundation of the stress reaction in newborn children and since there is only one other study concerning this topic our findings are promising with regard to further research. For instance, the statistically highest increase in MAOA LL homozygotes is interesting, taking into account the fact that childhood trauma (potentially leading to HPA dysregulation) has the worst impact (quantified by the amount of antisocial behavior) in male LL carriers (Caspi et al., 2002). The connection of the met allele with a higher cortisol decrease is again relatively pioneering considering the role of dopamine in HPA negative feedback and this hypothesis deserves scientific verification. And, last but not least, it would be interesting to follow the logic of testing the hypotheses of HPA axis reactivity as a mediator between genetic polymorphisms and temperament.

## CONFLICT OF INTEREST

None declared.

## Data Availability

The data that support the findings of this study are available from the corresponding author upon reasonable request.
